# Environment is associated with chytrid infection and skin microbiome richness on an amphibian rich island (Taiwan)

**DOI:** 10.1038/s41598-022-20547-3

**Published:** 2022-09-30

**Authors:** Dirk S. Schmeller, Tina Cheng, Jennifer Shelton, Chun-Fu Lin, Alan Chan-Alvarado, Adriana Bernardo-Cravo, Luca Zoccarato, Tzung-Su Ding, Yu-Pin Lin, Andrea Swei, Matthew C. Fisher, Vance T. Vredenburg, Adeline Loyau

**Affiliations:** 1 Laboratoire Ecologie Fonctionnelle et Environnement, Université de Toulouse, INPT, UPS, Toulouse, France; 2 Department of Biology, San Francisco State University, 1600 Holloway Ave, San Francisco, CA 94132 USA; 3 Bat Conservation International, Washington, DC USA; 4 Department of Infectious Disease Epidemiology, Imperial College London, London, W2 1PG UK; 5 Zoology Division, Endemic Species Research Institute, Jiji, Nantou Taiwan, ROC; 6 Department of Experimental Limnology, Leibniz-Institute of Freshwater Ecology and Inland Fisheries (IGB), Alte Fischerhütte 2, 16775 Stechlin, Germany; 7 School of Forestry and Resource Conservation, National Taiwan University, Taipei City, 106 Taiwan, ROC; 8 Department of Bioenvironmental Systems Engineering, National Taiwan University, Taipei, Taiwan, ROC; 9 Museum of Vertebrate Zoology, University of California Berkeley, Berkeley, CA 94720 USA

**Keywords:** Ecological epidemiology, Freshwater ecology, Microbial ecology, Environmental health

## Abstract

Growing evidence suggests that the origins of the panzootic amphibian pathogens *Batrachochytrium dendrobatidis* (*Bd*) and *Batrachochytrium salamandrivorans* (*Bsal*) are in Asia. In Taiwan, an island hotspot of high amphibian diversity, no amphibian mass mortality events linked to *Bd* or *Bsal* have been reported. We conducted a multi-year study across this subtropical island, sampling 2517 individuals from 30 species at 34 field sites, between 2010 and 2017, and including 171 museum samples collected between 1981 and 2009. We analyzed the skin microbiome of 153 samples (6 species) from 2017 in order to assess any association between the amphibian skin microbiome and the probability of infection amongst different host species. We did not detect *Bsal* in our samples, but found widespread infection by *Bd* across central and northern Taiwan, both taxonomically and spatially. Museum samples show that *Bd* has been present in Taiwan since at least 1990. Host species, geography (elevation), climatic conditions and microbial richness were all associated with the prevalence of infection. Host life-history traits, skin microbiome composition and phylogeny were associated with lower prevalence of infection for high altitude species. Overall, we observed low prevalence and burden of infection in host populations, suggesting that *Bd* is enzootic in Taiwan where it causes subclinical infections. While amphibian species in Taiwan are currently threatened by habitat loss, our study indicates that *Bd* is in an endemic equilibrium with the populations and species we investigated. However, ongoing surveillance of the infection is warranted, as changing environmental conditions may disturb the currently stable equilibrium.

## Introduction

Globalization and environmental change has led to the emergence of infectious diseases that threaten biodiversity and contribute to the ongoing 6th mass extinction^1,2^. Prominent examples are white-nose-syndrome in bats^3^, ash tree dieback^4^ and chytridiomycosis in amphibians^5^. Diseases caused by fungal pathogens such as these are emerging worldwide, leading to attrition in biodiversity, ecosystems and food security^6,7^. Declines due to epizootics that affect multiple host species simultaneously are leading to cascading effects across food webs through changes in species interactions and resulting ecosystem-level changes^8,9^.

Two emerging fungal pathogens, *Batrachochytrium dendrobatidis* (*Bd*) and *Batrachochytrium salamandrivorans* (*Bsal*), which cause the disease chytridiomycosis, have impacted amphibian populations around the world^10–12^. The geographic origin of *Bd* has been contested and the sources were variously suggested to be South Africa^13^, North America^14^, South America^15^, Japan^16^, and East Asia^17^. However, there is now compelling evidence that *Bd* may have originated from the Korean Peninsula^18^; this region is a global center of *Bd* genetic diversity^19^ and places the ancestral population of *Bd* within East Asia, corroborating results from China^20^, Korea^21,22^, Japan^16^, and Indonesia^23^. An out-of-Asia origin for *Bsal* has also been proposed based on the co-occurrence of these species in the region, and the lack of epizootics^11^. While *Bsal* has been found in East Asian countries^11^, predominantly in Vietnamese salamanders^24^, a widespread occurrence of *Bsal* has not been confirmed for China^20,25^ (but see^26^). Generally, no clear evidence for disease and declines associated with *Bd* or *Bsal* exist for Central Asia despite its potential role as source region of the most devastating infections ever recorded^26^.

The global trade in amphibians has led to ample opportunities for vectoring these pathogens worldwide^27^. While mass die-offs of amphibians have not been reported in Asia, our current knowledge about host range, environmental preferences, and distributions, as well as the potential impact of fungal pathogens in Asia is limited. In order to fill this knowledge gap, there is a pressing need to undertake surveillance of *Bd* and *Bsal* across their putative native range to more fully understand the factors that account for their distribution, epidemiology, and susceptibility of hosts^26,28^.

Identifying the biotic and abiotic factors that modify the prevalence of infection is necessary to better understand the interaction between hosts, pathogens, environment, and co-occurring microorganisms^29,30^. A large body of evidence supports the importance of symbiotic bacteria and skin defense peptides as defense mechanisms against pathogens including skin-infecting chytridiomycetes^31,32^. It has been shown that higher microbial richness and diversity can reduce the risk of *Bd* infection^30^. For example, laboratory infections have shown that bacterial diversity may influence whether *Bd* can infect an individual and to what intensity^33^. Under natural conditions, the skin microbiome of wild *Bd*-infected amphibians usually differs from uninfected individuals^34^. The differences were attributed to *Bd*-induced microbial dysbiosis, or a disruption of bacteria abundances^35^. We are also only at the beginning of our understanding of the factors that determine the structure of microbial communities on amphibian skin^36–39^. For instance, it is known that the amphibian skin microbiome is not wholly determined by the environmental microbial community, but also shows specificity to the host species^30,40–43^.

We surveyed wild amphibian populations in the tropical mountainous island of Taiwan, an important amphibian diversity hotspot in Asia. We sampled for the presence of *Bd* and *Bsal* and complemented our survey with formalin-preserved museum specimens collected between 1981 and 2009 to determine the historical presence of *Bd* in Taiwan. We analyzed species susceptibility, geography (elevation), microbial richness, and climatic conditions to determine chytrid infection dynamics in a potential source region of *Bd* and *Bsal*.

## Results

We did not find any evidence of *Bsal* infections in any of our samples (field and museum) and we found relatively low infection prevalence and infection intensities of *Bd*, with 182 positive individuals (7%) in the field across all years and species. From the 30 species sampled in the field, we found that 9 species (N = 426) did not show evidence of *Bd* infection across all years sampled, 12 species had a low infection prevalence (< 10%) and five species had a *Bd* prevalence equal to or above 10% (Supplementary Table 1). Excluding specimen with no evidence of infection, mean infection intensities were low with only three species showing mean infection intensities above 100 zoospore equivalents (ZE; Supplementary Table 1) and four species showing maximum infection intensities above 1000 zoospore equivalents (ZE; *Buergeria choui* = 1125 ZE; *Nidirana okinavana* = 4239 ZE; *Hylarana latouchii* = 90,000 ZE; *Limnonectes fujianensis* = 16,500 ZE). Across samples from 2010 to 2017, differences of *Bd* prevalence and infection intensities between Anurans (Prevalence = 0.090 ± 0.286; *Bd* ZE = 69.470 ± 2075.703) and Caudata (Prevalence = 0.045 ± 0.210; *Bd* ZE = 3.784 ± 37.998) were not significant (Prevalence: *U*_2090_ = 134,029; *p* = 0.084; *Bd* load: *U*_2090_ = 134,186; *p* = 0.076). However, when tested at the family level, prevalence and infection intensity significantly differed between the seven different amphibian families (Prevalence: *Χ*^2^_4_ = 57.85; *Bd* ZE: *Χ*^2^_4_ = 60.610; *p* < 0.001). This difference was attributed mostly to Rhacophoridae in comparison to other families (*Z*_*2090*_ = 3.829; *p* < 0.001) and the difference between Ranidae and Bufonidae (*Z*_*1456*_ = − 3.134; *p* = 0.015). Comparisons between years of sampling showed that in 2016 *Bd* prevalence was the highest (Supplementary Fig. 1), while a peak in *Bd* load was observed in 2013 (ZE_2013_ = 1070 ± 976; ZE_2010_ = 19 ± 7; ZE_2012_ = 0; ZE_2016_ = 895 ± 743; ZE_2017_ = 623 ± 499).

**Figure 1:**
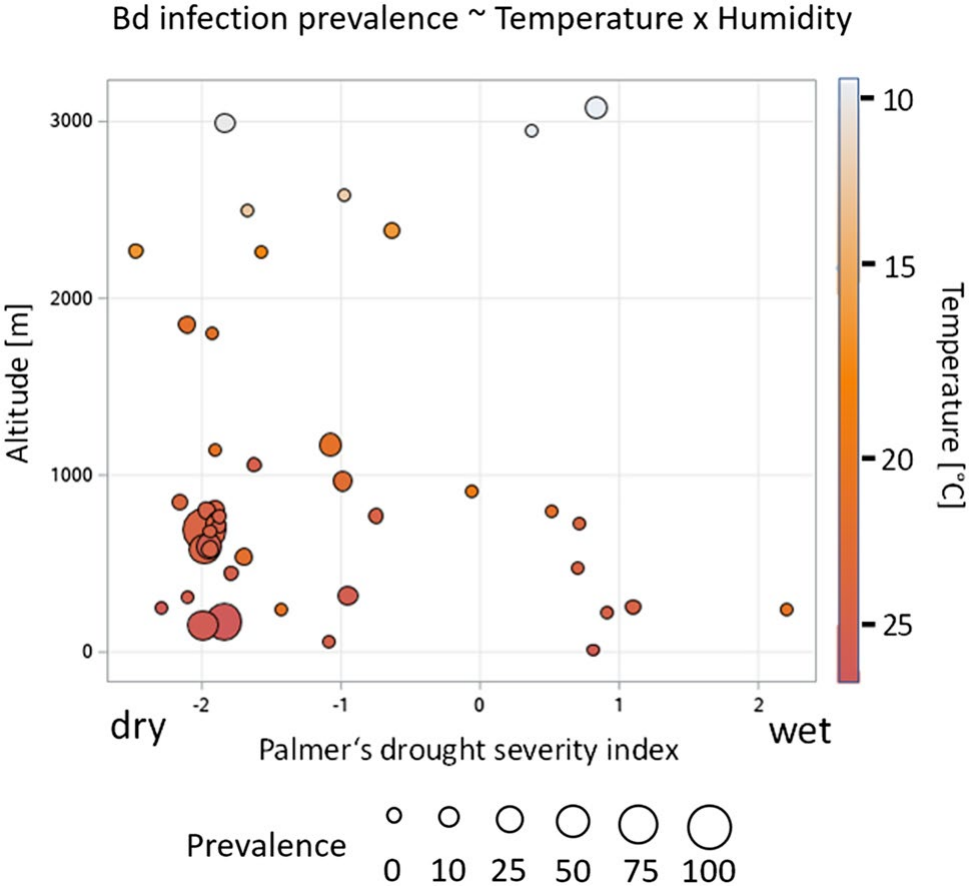
*Bd*-prevalence (%, bubble size, continuous) as a function of elevation, Palmers Drought Severity Index (PDSI), and yearly maximum temperature (T_max_; bubble color).

**Figure 2:**
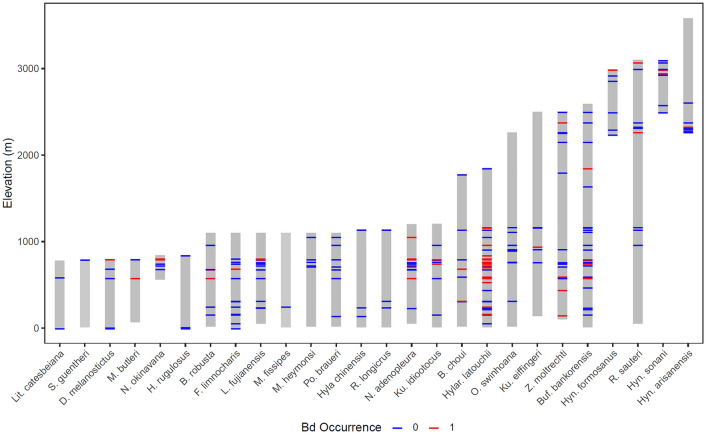
*Bd* occurrence by species and elevation. The grey bars represent the elevational range of a species. Horizontal lines show at which elevation the species was sampled and whether *Bd* was found in that species at that elevation (blue = 0, *Bd* does not occur; red = 1, *Bd* occurs).

We found that the best model to explain *Bd* prevalence and *Bd* load included genus, year, elevation, Palmer Drought Severity Index (PDSI) and the interaction of elevation or PDSI and genus (Table 1). Models including any of the temperature variables had lower AICs (Akaike’s Information Criterion; Table 1). Overall, our results suggest that PDSI and the interaction of PDSI and elevation are important drivers of *Bd* prevalence and load, with infected sites characterized by drier conditions and located in intermediate elevations. Elevation also contributes to explaining *Bd* load, but not *Bd* prevalence (Table 1). Results of the models including temperatures show that yearly mean temperature (T_mean_) poorly explains *Bd* prevalence and load (in terms of AICs and *p*-values), compared to temperature extremes (yearly maximum temperature T_max_; and yearly minimum temperature T_min_) and their interactions with PDSI (Table 1). Drier habitats, and generally higher temperatures led to higher infection probability (Fig. 1). The different taxa show differences in their altitudinal distribution (Fig. 2), and the interaction between genus and elevation was a good predictor for *Bd* prevalence (*F*_14, 2043_ = 3.49; *p* < 0.001) and *Bd* load (*F*_14, 2043_ = 3.55, *p* < 0.001).

**Table 1:** Model results of the generalized linear mixed model analysis with *Bd* prevalence and *Bd* load as dependent variables and genus, year, elevation, Palmers Drought Severity Index (PDSI), yearly minimum (T_min_), mean (T_mean_), or maximum (T_max_) temperature as explanatory variables.

Model rank	Explanatory variable	Num DF	Den DF	*Bd* prevalence				*Bd* load			
				*F*	*p*	AIC	Δ_i_	*F*	*p*	AIC	Δ_i_
1	Genus	10	2043	4.07	< 0.0001	947.94	0	3.53	0.0001	1335.00	0
	Year	1	2043	21.07	< 0.0001			24.65	< 0.0001		
	Elevation	1	2043	0.03	0.8736			4.89	0.0271		
	PDSI	1	2043	44.07	< 0.0001			17.18	< 0.0001		
	Elevation*PDSI	1	2043	9.57	0.0020			8.18	0.0043		
	Elevation*Genus	10	2043	3.49	0.0001			3.55	0.0001		
2	Genus	10	2043	3.19	0.0004	954.28	6.34	3.19	0.0004	1344.39	9.39
	Year	1	2043	5.17	0.0230			8.80	0.0030		
	T_max_	1	2043	2.95	0.0861			2.07	0.1505		
	PDSI	1	2043	6.97	0.0084			4.83	0.0281		
	T_max_*PDSI	1	2043	15.06	0.0001			6.46	0.0111		
	T_max_*Genus	10	2043	3.12	0.0006			3.39	0.0002		
3	Genus	10	2043	2.83	0.0017	962.27	14.33	3.30	0.0003	1346.37	11.37
	Year	1	2043	12.30	0.0005			17.49	< 0.0001		
	T_min_	1	2043	5.99	0.0145			5.00	0.0255		
	PDSI	1	2043	2.84	0.0919			3.75	0.0530		
	T_min_*PDSI	1	2043	12.31	0.0005			6.38	0.0116		
	T_min_*Genus	10	2043	3.16	0.0005			3.72	< 0.0001		
4	Genus	10	2043	1.59	0.1032	980.28	32.34	1.49	0.1356	1372.16	37.16
	Year	1	2043	23.70	< 0.0001			16.99	< 0.0001		
	T_mean_	1	2043	1.71	0.1907			0.00	0.9546		
	PDSI	1	2043	0.18	0.6704			1.27	0.2597		
	T_mean_*PDSI	1	2043	1.44	0.2301			1.94	0.1635		
	T_mean_*Genus	10	2043	1.32	0.2150			1.41	0.1697		

*DF* degrees of freedom, *AIC* Akaike’s Information Criterion, *Δ*_*i*_ difference to best model.

In our museum samples (N = 171), we found five *Bd* positive individuals (prevalence = 3%), four of which were salamanders (2 specimens each of *Hynobius ariasanensis* (2001) and *Hynobius formosanus* (1994)) and only one was an anuran (*Rana sauteri*) from 1990 (Supplementary Table 2).

**Figure 3:**
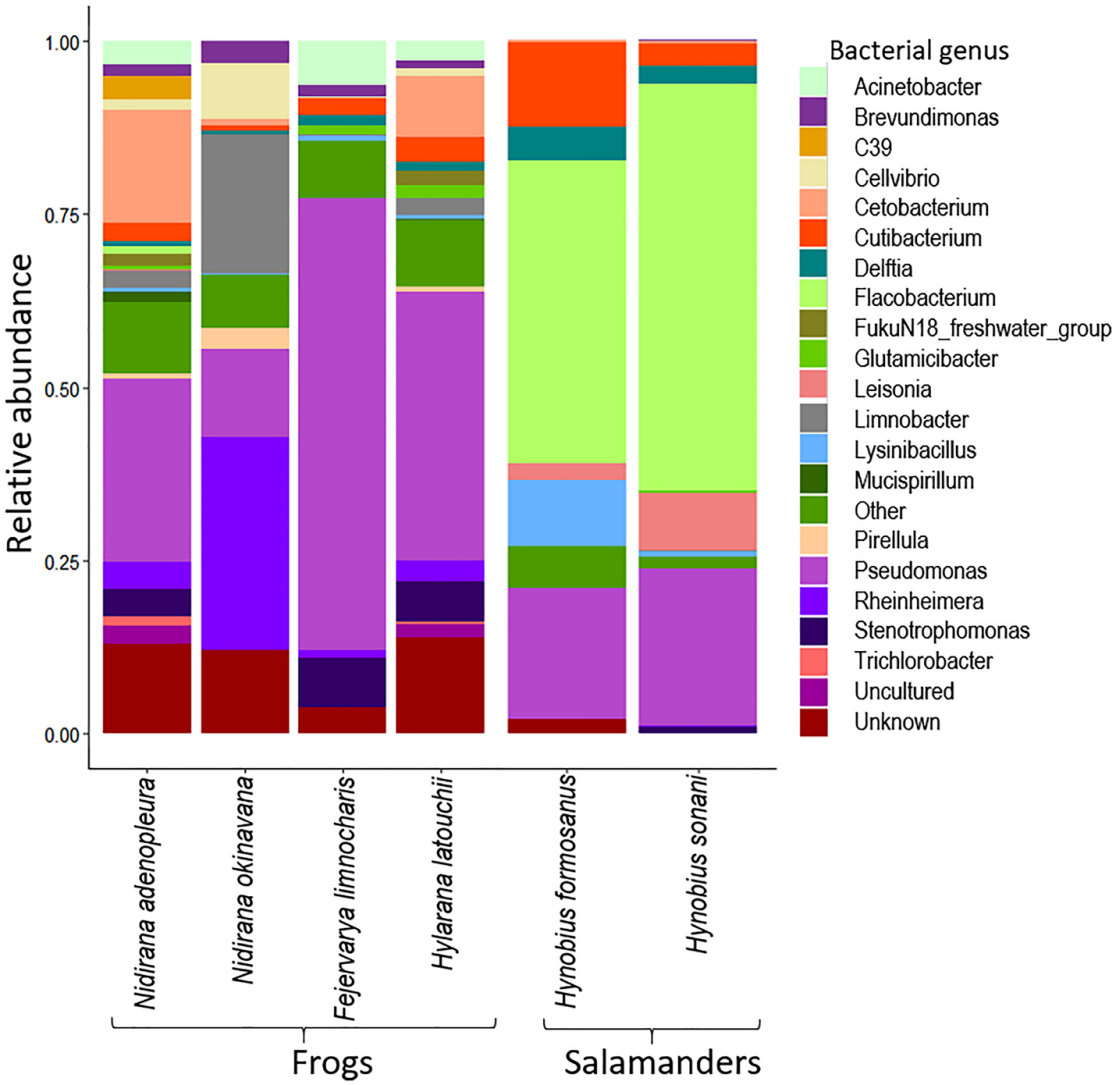
The skin microbial communities of the core bacterial genera occurring in at least 75% of individuals of four species of frogs and two species of salamanders.

**Figure 4:**
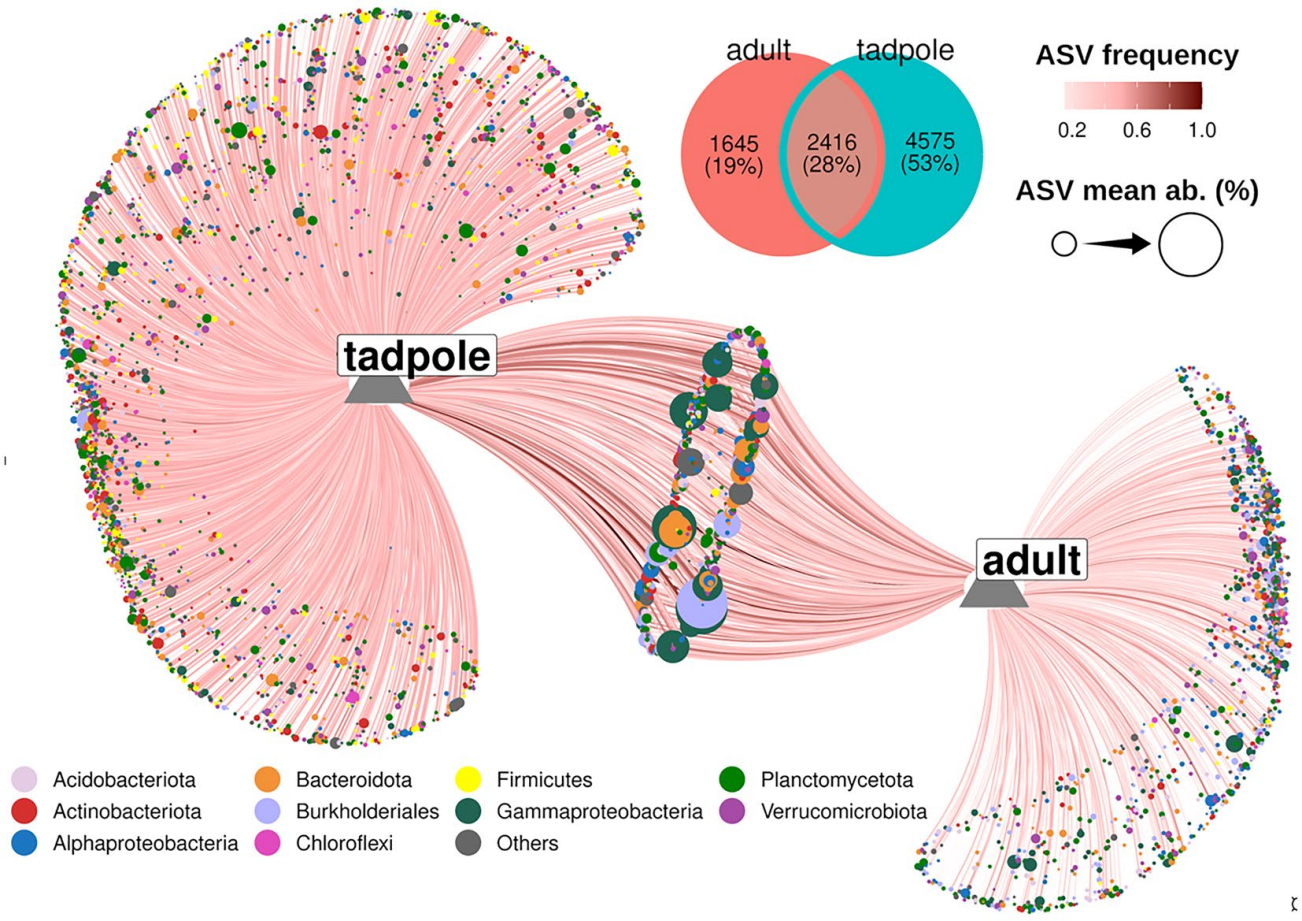
Skin microbiome composition of *N. adenopleura* at the tadpole (n = 18) and adult (n = 13) life stages caught at the same time in the same location and habitat. Only ASVs occurring in at least 3 different samples are visualized in the network. The size of each node corresponds to the mean relative abundance of an ASV in the considered samples, while the colour indicates the ASV’s taxonomic affiliation. The node size corresponds to ~ 4% for the largest circles to ~ 0.001% of the smallest ones (i.e. larger nodes are shared and smaller are unique but also highly variable among individuals). The Venn diagram shows the size of the unique and shared node sets.

We observed a marked difference of the skin microbiome of adult frogs as compared to salamanders (*F*_1,90_ = 27.149, *p* < 0.001) and between species of frogs (*F*_3,54_ = 9.286, *p* < 0.001; Fig. 3), as well as an effect of life stage (tadpole vs. adult) on the skin microbiome of *N. adenopleura* (*F*_1,67_ = 13.946, *p* < 0.001). In *N. adenopleura* we observed that adults and tadpoles possessed a unique set of Amplicon Sequence Variants (ASVs), with a richer microbiome in tadpoles (N_ASVs_ = 4575) as compared to adults (N_ASVs_ = 1645). However, many of the ASVs unique to the two life stages had low abundance, while the most common ASVs from *Gammaprotobacteria*, *Burkholderiales* and *Bacteroidota* were shared between adults and tadpoles at the same time and site (Fig. 4). On salamanders, the most common genera (occurrence in 75% of samples) were *Flavobacterium*, *Pseudomonas*, and *Cutibacterium*, with only 5 core ASVs (4 of *Flavobacterium* and one of *Cutibacterium*). On adult frog skin, we observed very few *Flavobacteria*. The most common genera across all four species of frogs included *Cutibacterium*, *Pseudomonas*, *Delftia*, *Blastococcus*, *Rheinheimera*, and *Lysinibacillus* (Fig. 3).

Our data does not support an effect of isolation-by-distance on the skin microbiome of both adult frogs (*r*_418_ = 0.242; *p* = 0.053) and tadpoles (*r*_259_ = 0.024; *p* = 0.386), but site was an important determinant of adult frog skin microbiome composition (permanova *F*_4,53_ = 27.785, *p* < 0.001).

**Figure 5:**
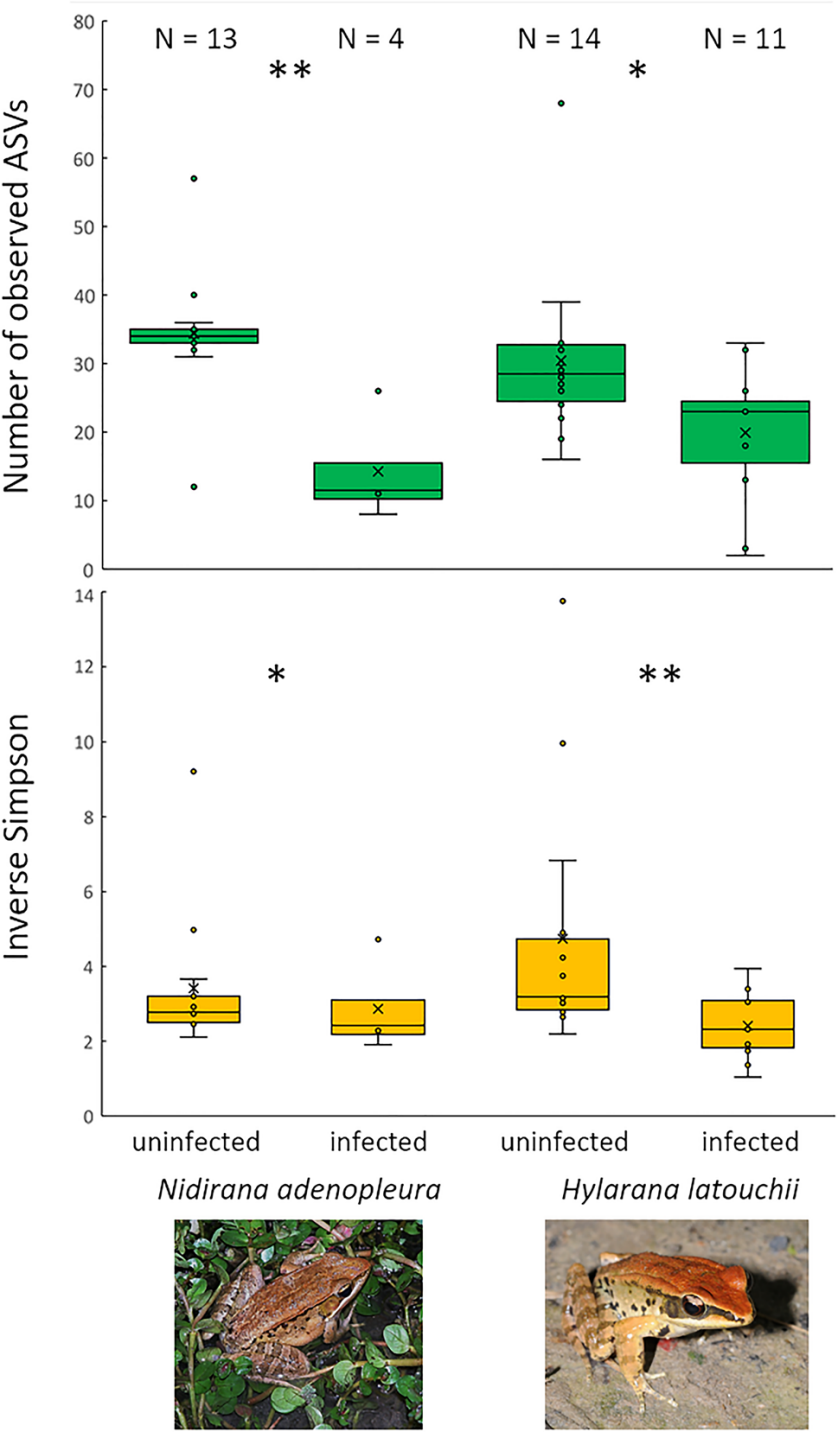
Comparison of species richness (number of observed ASVs) and evenness (inverse Simpson index) of infected and uninfected specimens of *N. adenopleura* and *H. latouchii*. We observed a significantly higher richness (N_ASVs_, *N. adenopleura*: *U*_16_ = 50.5, *p* = 0.003, *H. latouchii*: *U*_24_ = 116, *p* = 0.031) and evenness (Inverse Simpson, *N. adenopleura*: *U*_16_ = 47.0, *p* = 0.015; *H. latouchii*: *U*_24_ = 124, *p* = 0.009) in uninfected individuals as compared to infected individuals.

For *N. adenopleura* and *H. latouchii*, we compared alpha diversity indices and observed a significantly higher richness and evenness in uninfected individuals (Fig. 5). The permanova analysis confirms a significant difference of the skin microbiome in infected vs. uninfected adults for *N. adenopleura* (*F*_1,15_ = 4.385, *p* = 0.003) and *H. latouchii* (*F*_1,23_ = 3.752, *p* = 0.009) with higher richness and evenness leading to lower infection probability (Fig. 5). The indicator analysis on the ASV level reveals that both frog species have in common *Pseudomonas* ASV 4, and *Stenotrophomonas* ASV 7 in infected specimens, and *Limnobacter* ASV 31 and *Cetobacterium* ASV 1 in uninfected specimens (Supplementary Fig. 2).

Across all species and life-stages, species evenness, but not richness, is explained by climatic variables and elevation (Inverse Simpson, T_max_: *F*_1,149_ = 7.59, *p* = 0.007; PDSI: *F*_1,149_ = 4.93, *p* = 0.028) and their interaction (*F*_1,149_ = 6.79, *p* = 0.010). Considering adult frogs only, species richness and evenness were both significantly linked to temperature, PDSI and their interaction (Table 2). The relationship is not driven by *Bd*-induced dysbiosis, as it holds true for uninfected frog adults only and the relationship of evenness with T_max_ (*F*_1,39_ = 6.70, *p* = 0.013), PDSI (*F*_1,39_ = 6.54, *p* = 0.015) and the interaction of T_max_ and PDSI (*F*_1,39_ = 6.84, *p* = 0.013). Generally, microbial species richness and evenness were higher at sites with colder and drier climate conditions.

**Table 2:** Model results of the generalized linear mixed model analysis with species richness (number of observed ASVs) and evenness (inverse Simpson index) as dependent variables and elevation, Palmers drought severity index (PDSI), yearly minimum (T_min_), mean (T_mean_), or maximum (T_max_) temperature as explanatory variables.

Model rank	Explanatory variable	Num DF	Den DF	Richness				Evenness			
				*F*	*P*	AIC	Δ_i_	*F*	*p*	AIC	Δ_i_
1	T_max_	1	54	5.09	0.0281	113.94	0	10.19	0.0024	− 27.90	0
	PDSI	1	54	5.03	0.0290			9.94	0.0026		
	T_max_*PDSI	1	54	5.10	0.0280			10.31	0.0022		
2	T_mean_	1	54	5.04	0.0289	114.20	0.26	9.58	0.0031	− 27.65	0.25
	PDSI	1	54	4.94	0.0304			9.19	0.0037		
	T_mean_*PDSI	1	54	5.05	0.0287			9.74	0.0029		
3	T_min_	1	54	4.94	0.0305	114.41	0.47	8.86	0.0044	− 27.44	0.46
	PDSI	1	54	4.78	0.0331			8.25	0.0058		
	T_min_*PDSI	1	54	4.97	0.0300			9.07	0.0040		
4	Elevation	1	54	4.53	0.0379	139.65	25.71	8.94	0.0042	− 3.05	24.85
	PDSI	1	54	5.62	0.0214			16.09	0.0002		
	Elevation*PDSI	1	54	4.55	0.0374			9.13	0.0038		

*DF* degrees of freedom, *AIC* Akaike’s Information Criterion, *Δ*_*i*_ difference to best model.

## Discussion

Here, we investigated the presence of the fungal pathogens *Bd* and *Bsal* in amphibians of Taiwan, an island of high amphibian diversity close to the putative region of endemism for both *Bd*^18^ and *Bsal*^26^. We did not detect *Bsal* in any of our samples, however, it is likely that the global panzootic lineage of *Bd* (*Bd*-GPL) is potentially widespread in Taiwan, both taxonomically and spatially, based on whole genome sequencing of representative isolates from the island^18^. Analysis of museum samples suggest that *Bd* has been present in Taiwan since at least 1990. The pattern of *Bd* infection appears to be influenced by host species susceptibility, geography (elevation), climatic conditions (temperature extremes and dryness) and host skin microbiome composition. Our study suggests that alpha-diversity on amphibian skin was driven by climatic conditions and landscape context and not by *Bd*-driven dysbiosis.

Our data suggest that environmental conditions, such as elevation, dryness and temperature are important factors determining the prevalence of infection, but the interaction with host-specific properties also appear to be important. *Bd* infection dynamics are complex and multiple factors may affect the outcome^44^. Our study suggests that *Bd* was more commonly found on hosts that live at medium elevation (1000–1600 m a.s.l), at temperatures up to 25 °C and drier sites. In such habitats, anurans in the families Ranidae and Rhacophoridae are particularly numerous, suggesting that life-history traits, i.e. habitat preferences and aquatic larval stages of these host species may modulate *Bd*-dynamics. Generally, the relationship between *Bd* infection, amphibian hosts and temperature is not yet clearly understood^45^. In culture, *Bd* develops particularly well between 17 and 23 °C and dies at temperature above 29 °C and below freezing^46,47^. Under natural conditions *Bd* growth and infection is also related to the temperature dependent amphibian immune system^48^ and, as our study suggests, to the level of environmental humidity. In the warm temperate and mesic environmental zone, we found a high number of different host species as well as the most highly infected individuals and the highest *Bd* prevalence. In the lowland regions of Taiwan, where we found the least *Bd* infected hosts, temperatures can easily exceed the maximum temperature of *Bd* (29 °C), and even if individuals get infected, they might be able to clear *Bd* infections due to those high temperatures^49^. At higher elevations, host diversity and density are strongly reduced, and most of the species (most of them salamanders) are less aquatic. The adults are mainly terrestrial and although they lay eggs in nearby streams, they typically do not aggregate in breeding habitats for long periods of time. These life-history traits may lead to lower infection probabilities^50^ and may explain why we found the interaction between genus and elevation can help predict *Bd* prevalence and load on hosts. Temperatures continue to rise in Taiwan and elsewhere, and thus an upward elevational upward shift of *Bd* prevalence is predicted.

The already complex interactions of *Bd*, host, and environment might additionally be modulated by the host skin microbiome, as suggested by the recently proposed disease pyramid^30^. The climatic conditions defined by temperature and humidity are correlated with the richness and diversity of amphibian skin microbiomes^38^. We observed higher diversity and evenness of the skin microbiome in uninfected vs. infected individuals, and we suggest that temperature may not only have a direct impact on the host’s inner immune system and *Bd* growth, but also on the exterior host immune system formed by the skin microbiome^30^. Given that, in the Olive frog, the most common ASVs are shared between adults and tadpoles, the interactions between temperature and skin microbiome may be very similar in the different life stages. To understand the functional causes for differences in infected vs. uninfected individuals, and to predict the outcome for Taiwan’s amphibian diversity from the interaction of *Bd*, host, host microbiome, and climate change more data and more microbial research are needed.

One of the prevailing questions that remains regarding *Bd* is whether this pathogen is emerging, persisting, or endemic in Taiwan. *Bd* was found in a specimen of *Adrianus japonicus* collected in 1902 in Japan, and thus suggests that *Bd* is endemic in Japan^16^. Unlike other parts of the world that have experienced *Bd*-associated declines, there have not been any documented reports of enigmatic declines in Asian amphibian populations. In Taiwan, there has been no documentation of enigmatic declines in native amphibians, barring one report of a mass mortality event in *R. sauteri* that occurred in 1995 at the Sitou Forest Recreation Area (Chun-Fu Lin, personal comm.). During that event, around thirty individuals were found dead on the edge of a stream. Individuals exhibited red legs and skin lesions, symptoms associated with multiple pathogens, such as ranaviruses^51^ or infection by *Aeromonas hydrophila*, but not with *Bd*. Despite the presence of *Bd* in wild amphibian populations, we observed few individuals exhibiting disease symptoms characteristic of chytridiomycosis (tadpoles with missing mouthparts, lethargy, excessive skin sloughing, leg-locking, loss of righting reflex, etc.), with only one highly lethargic *H. latouchii* male exhibiting clinical signs associated with chytridiomycosis (ZE = 3.29). Generally, *Bd* was usually found at low levels of infection (less than 100 ZE) and with low prevalence in populations, which indicates that *Bd* is likely enzootic in Taiwan. Our method, however, does not allow to know if the global pandemic lineage of *Bd* (*Bd*-GPL) has been present in Taiwan before 1990 or if the less virulent lineage of *Bd*-Asia or unknown *Bd* lineages were present before, enabling the Taiwanese amphibian fauna to preadapt to *Bd*-GPL.

Even though *Bd* likely is enzootic in Taiwan, it may still drive population declines at low levels of disease prevalence^52^, likely also due to ongoing environmental changes such as climate change or pollution. Of greatest concern for conservation and *Bd* monitoring are those species that are endemic and endangered in Taiwan. Amphibians in this category that tested positive for *Bd* are salamanders within the family Hynobiidae (*H. sonani*, *H. arisanensis*, *H. formosanus*), usually limited to a specific mountain region of the Zhong Yang mountain range. However, only very few individuals tested positive for *Bd* and prevalence was well below 10%, indicating enzootic infection levels. Other threatened species found to be infected were *Rana sauteri*, and the highly endemic species of *N. okinavana* known only from three locations globally, of which two sites (one location) are found in Taiwan. Despite small sample sizes for these rare species, *Bd* was detected, but prevalence was low. However, the impact that *Bd* is having on these species is largely unclear, due to very limited data on population dynamics, currently not allowing population viability analyses. Low infection levels found in these individuals may indicate that they are persisting with low levels of infection, and may have achieved natural immune defenses against the fungus from a longer co-evolutionary interaction, but more intensive disease and population monitoring is needed.

Our study investigated multiple dimensions of disease dynamics in a poorly-studied yet diverse system that harbors *Bd* in an area of the world that has not experienced *Bd* epizootics. This information should help us better understand how the *Bd* pathogen is maintained in an enzootic state in multi-host systems. Our study further stresses that the *Bd*-amphibian disease system is highly complex^44^ and only a holistic research approach is able to unravel all the factors modulating infection outcome and disease expression, including micropredators^53^, pollution^29^, climate^44^, and microbial communities^36,38^.

## Materials and methods

### The island of Taiwan

**Figure 6:**
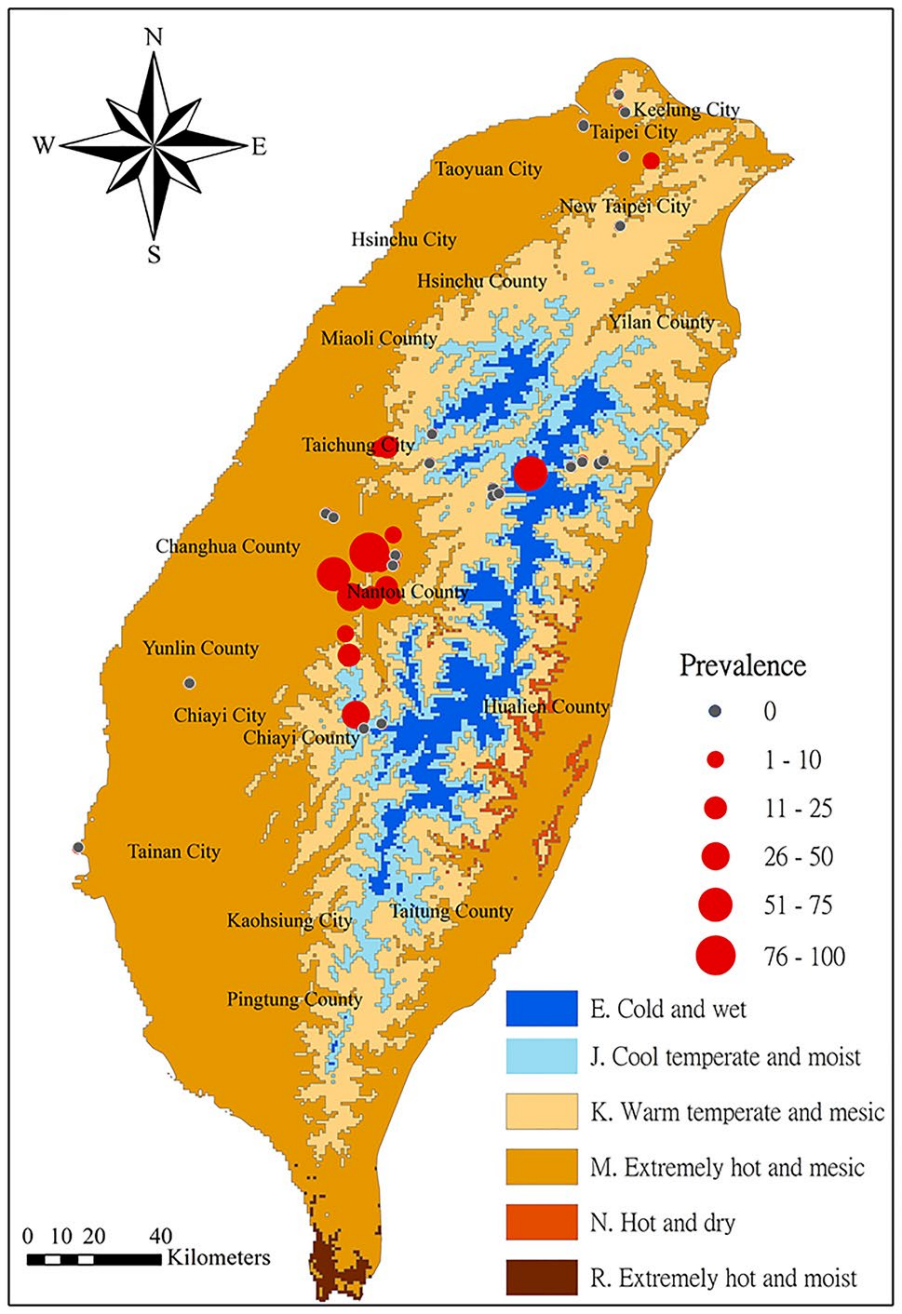
Distribution of *Bd* in the different bioclimatic zones of Taiwan^55^. The circle size corresponds to the prevalence class.

The main island of Taiwan has a humid tropical climate and is characterized by 6 environmental zones^54^ (Fig. 6). The northern part around the city of Taipei, the low mountain range and the western plain receive variable precipitations based on season. Temperatures in the lowlands usually do not get below 5 °C, and the weather in summer is tropical and very humid, especially in mid to lower elevations. Lower temperatures and humidity can be observed between November and April and maximum temperatures around 38 °C can be observed during June to August. In some cases, there is frost or snow on the high mountains between January and March. The high relief of Taiwan and the climatic conditions lead to a high diversity of different habitats that span from lowlands to high elevations. These diverse environmental conditions gave opportunity for amphibians to fill a large range of different ecological niches, making Taiwan an important amphibian diversity hotspot with at least 41 currently known amphibian species, of which many have very restricted distribution areas (Amphibiaweb.org). The warm temperate and mesic zone, which contains mountains of low to medium elevation (500–1500 m above sea level), is the most amphibian rich zone of Taiwan. Thus, Taiwan is an ideal region to investigate climatic variables influencing the occurrence and prevalence of *Bd* and *Bsal* on amphibians.

### Study sites and field sampling

We sampled 26 of the 41 currently known Taiwanese amphibian species (total N = 2517 individuals) across northern and central Taiwan in July/August 2010 (N = 328 individuals), February 2012 (N = 440), September 2013 (N = 373), April 2016 (N = 672) and May 2017 (N = 704). We obtained samples from different environmental zones (Fig. 6).

When possible, we sampled the same location over different years and aimed for 30 samples per species, life stage and location to ensure a 95% chance of detecting the pathogen at a prevalence of 5%^56^. All individuals were hand-caught and sampled by swabbing the ventral surface of adults and mouthparts of tadpoles with a synthetic cotton swab (Medical Wire MW-100) for *Bd* and *Bsal* analyses, and released at site of capture. *Bd* and *Bsal* swabs were stored in a cool box with cool packs and transferred to a refrigerator until the DNA was extracted.

In addition, in 2017 we took skin microbiome samples from up to 10 tadpoles and 10 adults per site by rubbing sterile dry swabs across the whole body surface. The microbiome swabs were immediately frozen at − 79 °C using dry ice and transferred to a freezer at earliest convenience (− 30 °C) until DNA extraction. We analysed skin microbiomes in a total of 153 samples: 34 salamander specimens (22 *H. sonani*, 12 *H. formosanus*), 58 adult frogs (17 *N. adenopleura,* 9 *N. okinavana*, 7 *F. limnocharis*, 25 *H. latouchii*), and 61 tadpoles (51 *N. adenopleura*, 10 *H. latouchii*).

We also sampled formalin-preserved specimens from the National Museum of Natural Science in Taichung, Taiwan, that were collected between 1986 and 2009, and formalin-preserved hynobiid salamanders from the lab of Dr. June-Shiang Lai at National Taiwan Normal University, Taipei, collected between 1981 and 2009 (N species = 7; N individuals = 171, Supplementary Table 2). Museum samples were first rinsed thoroughly with ethanol and swabbed with synthetic cotton swabs (Medical Wire MW-100) following established protocols^57,58^.

We did not conduct in-vivo experiments and our sampling protocol was not invasive, following national Taiwanese standards of animal wellbeing in force (Taiwan Animal Protection Act, Taiwan Wildlife Conservation Act) at the time of capture (amendments have been done in May 2021). As we did not use drugs, traps, firearms, poison or corrosive substances to capture animals, the Animal Protection Act of Taiwan does not oblige the calling on an ethics committee at the time of sampling. Further, the Taiwan Endemic Species Research Center (Chun-Fu Lin) is an officially authorized and commissioned organization within the Taiwan Wildlife Conservation Act to conduct all necessary work to protect wild species, including disease monitoring. All animals were released at their original site unharmed.

### Genetic analysis and qPCR assays

DNA was extracted from swabs with commercially available kits (Nucleospin, Prepman). A cost-effective Prepman extraction was used for the chytrid pathogen extractions^59^ and Nucleospin kits were used for extraction of the microbiome as this captures the fungal part of the microbiome better.

For determination of *Bd* and *Bsal* prevalence and infection loads, extracts were diluted 1:10 in 0.25xTE Buffer and run using the Taqman real-time PCR protocol following previously published protocols^60,61^. In order to achieve a final infection intensity level measured in terms of zoospore equivalents (ZE), qPCR genomic equivalent (GE) values were multiplied by 40 to account for dilution during extraction. We report *Bd* loads as mean ZE_swab_ ± SE, considering all ZE values above zero as positive. We define prevalence of infection as [(no. infected/no. sampled) * 100].

Skin bacterial communities were characterized using 16S amplicon sequencing. DNA was extracted from swabs using the PowerSoil DNA Isolation Kit (MoBio Laboratories, Carlsbad, CA, USA), and the hypervariable V3–V4 region of the bacterial 16S rRNA gene was amplified in triplicate using primers with overhang adaptors. Each 25-μL reaction consisted of 12.5 μL KAPA HiFi HotStart ReadyMix (KAPA Biosystems, Wilmington, MA), 5 μL forward and reverse primers (1 μM), and 2.5 μL template. PCR conditions were 95 °C for 180 s, followed by 25 cycles of 95 °C for 30, 60 °C for 15 s, 72 °C for 45 s, and a final extension of 72 °C for 120 s. The PCR products for triplicate reactions were pooled and purified using solid phase reversible immobilization (SPRI) beads (Agencourt AMPure XT, Agencourt Bioscience Corporation, Beverly, MA). All samples and negative control were visualized using gel electrophoresis. Dual indices, from the Illumina Nextera Index Kit, were attached to the purified amplicons using PCR. Each 25-µL reaction consisted of 12.5 µL Kapa HiFi HotStart ReadyMix, 2.5 µL forward and reverse primers (1 µM), 5 µL PCR-grade water, and 5 µL template. PCR conditions were 95 °C for 180 s, followed by 10 cycles of 95 °C for 30 s, 55 °C for 30 s, 72 °C for 30 s, and a final extension of 72 °C for 300 s. The PCR products were purified and visualized as described above. DNA concentrations were quantified using Qubit Fluorometric Quantification and samples were diluted and pooled at equimolar concentrations. Sequencing was performed on an Illumina MiSeq using a MiSeq Reagent Kit v3 (600 cycle) (Illumina, Inc., San Diego, CA).

Base calling and demultiplexing were performed using MiSeq Reporter (Illumina, Inc.), while removal of primer and adapter sequences was performed using Cutadapt^62^. Additional quality filtering and trimming (truncLen = c(260, 200), maxEE = c(5, 5)), formation of contiguous sequences (minOverlap = 12), identification of unique amplicon sequence variants (ASVs; pool = "pseudo") and chimera removal were performed using the DADA2 pipeline in R using default settings unless specified^63^. Taxonomy was assigned to ASVs using a local installation of SINA v1.7.2^64^ and SILVA 138.1^65^ release taxonomy. Samples were filtered out if they contained less than 100 ASVs. ASVs that were present in less than 0.001% of all reads and fewer than 2 samples were considered low quality and removed^66^. Samples were not rarefied, since rarefaction implies in loss of information and precision when defining a common read depth^67^. Data was normalized before the microbiome analysis.

### Climatic and environmental variables

We sourced data from TerraClimate of Climatology Lab^68^, spanning our full sampling period from 1980 to 2017. We obtained yearly mean temperature (T_mean_), yearly maximum temperature (T_max_) and yearly minimum temperature (T_min_). From the TerraClimate data set, we also obtained Palmers drought severity index (PDSI) data^69^, which uses readily available temperature and precipitation data to estimate relative dryness. It is a standardized index that spans from − 10 (dry) to + 10 (wet)^69^.

PDSI across our sampling sites has not changed over time, while the temperature has increased steadily over the years. The lowest maximum temperature observed was 20.07 °C in 1984, while the highest was observed in 2016 (21.45 °C). The year 1998 marks a first-time maximum temperature above 21 °C (21.43 °C), while the year 2012 marks the last time of a maximum temperature below 21 °C (20.55 °C). The lowest minimum temperature of 12.88 °C across our sites was observed in the year 1986, while the highest minimum temperature of 14.44 °C was observed in 1998. The driest years (PDSI < − 3.0) were 1993 (− 3.066), 1996 (− 3.272) and 2004 (− 3.824). The wettest years (PDSI > 3.0) were 1983 (4.690), 1998 (4.312) and 2001 (3.187). PDSI was not correlated with elevation (*R*_42_ = − 0.163, p > 0.05). All temperature variables and the elevation were highly intercorrelated (all *R* ≥ 0.611; all *p* < 0.01).

### Statistical analyses

We performed statistical analyses using SAS v.9.4. First, we used the full dataset of field samples (N = 2518) to compare pathogen prevalence and infection load between the two orders (Anura vs. Caudata), seven families (Table 2) and five years of sampling, using non-parametric tests including an U-test for the pairwise comparison of Orders, and a Kruskal–Wallis-ANOVA for k variable comparisons for the comparisons between families and between years of sampling (2010–2017).

We examined pathogen prevalence and infection intensity in our field samples with GLMMs (SAS proc glimmix). We discarded all data of species with no *Bd* detection over the years, assuming that they might not be susceptible to *Bd* (N = 13). This selection left us with data from 17 species (11 genera), and a sample size of 2090 specimens (Table 2). We built a first set of GLMMs with *Bd* prevalence (Prev) as the dependent variable (with a binomial distribution of error terms and a logit link function), and a second set of models with log-transformed infection loads (log(ZE)) as the dependent variable (with negative binomial distribution of error terms and a logit link function). The independent variables included in the models were genus, year of sampling, PDSI, and a temperature/elevation parameter. We also included the interactions of PDSI or genus with temperature/elevation. Due to collinearity between elevation and temperature, we constructed separate models with T_mean_, T_max_, T_min_ or elevation, and then used the AIC to rank two sets of four models.

We used permutational multivariate analyses of variance (permanova) to compare the bacterial communities between the same groups of samples (order, species, infection status). For the permanova analysis, the ASV abundance was transformed to compositional data and the Bray–Curtis dissimilarity distance was calculated with the *vegan* R-package. We performed LDA Effect Size (LEfSe) analysis (LDA score ≥ 2, p ≤ 0.05)^70^ using the LEfSe online tool (http://huttenhower.sph.harvard.edu/galaxy/) to compare indicator species (ASVs) between two groups (infected vs. uninfected specimens). We performed a Mantel test (*vegan* R-package) between the matrix of the geographic distance between the sites (Haversine distance based on latitude and longitude) and the matrix of Bray–Curtis dissimilarity based on the mean relative abundance of the bacterial genera.

We computed species richness as number of observed ASVs, and species evenness using the inverse Simpson index. We z-transformed ASVs and log-transformed the inverse Simpson index to yield a normal distribution. We investigated the impact of the climate variables T_max_, PDSI, and their interaction on species richness and evenness using GLMMs (SAS proc Glimmix), assuming normal distribution and using the identity link function.

To compare alpha diversity indices (observed ASVs, inverse Simpson) between infected and uninfected individuals we used the non-parametric Mann–Whitney U-test, due to the low number of infected individuals in our dataset. We compared skin microbiomes of the tadpole and adult stages of *N. adenopleura* using a network analysis. We picture the distribution of unique and shared prokaryotic ASVs among the two life stages. The network analysis was done using the R-packages network, ggnetwork and ggVennDiagram.

## Supplementary Information

Supplementary Information.
