# Supplementary material for: Environment is associated with chytrid infection and skin microbiome richness on an amphibian rich island (Taiwan)
Source: Sci Rep. 2022 Sep 30;12:16456. doi: 10.1038/s41598-022-20547-3 (PMC9525630; doi:10.1038/s41598-022-20547-3)
Supplement: Supplementary file 1 — Supplementary Information. [file 41598_2022_20547_MOESM1_ESM.docx]

**Supplementary Material to**

**Environment is associated with chytrid infection and skin microbiome richness on an amphibian rich island**

**Dirk S. Schmeller^1,*^, Tina Cheng^2,3^, Jennifer Shelton^4^, Chun-Fu Lin^5^, Alan Chan-** **Alvarado^2^, Adriana Bernardo-Cravo^1^, Luca Zoccarato^6^, Tzung-Su Ding^7^, Yu-Pin Lin^9^, Andrea Swei^2^, Matthew C. Fisher^4^, Vance T. Vredenburg^2,10,#^, Adeline Loyau^1,6,#^**

1. Laboratoire écologie fonctionnelle et environnement, Université de Toulouse, INPT, UPS, Toulouse, France
2. San Francisco State University, Department of Biology, 1600 Holloway Ave, San Francisco, CA USA 94132
3. Bat Conservation International, Washington (DC), USA
4. Department of Infectious Disease Epidemiology, Imperial College London, London W2 1PG, UK
5. Zoology Division, Endemic Species Research Institute, Jiji, Nantou, Taiwan
6. Department of Experimental Limnology, Leibniz-Institute of Freshwater Ecology and Inland Fisheries (IGB), Alte Fischerhütte 2, Stechlin, D-16775, Germany
7. School of Forestry and Resource Conservation, National Taiwan University, Taipei City, 106, Taiwan
8. Department of Life Science, National Taiwan University
9. Department of Bioenvironmental Systems Engineering, National Taiwan University
10. University of California Berkeley, Museum of Vertebrate Zoology, Berkeley, CA 94720

*Corresponding author: Adeline Loyau, al@adeline-loyau.net.

^#^ shared senior authorship


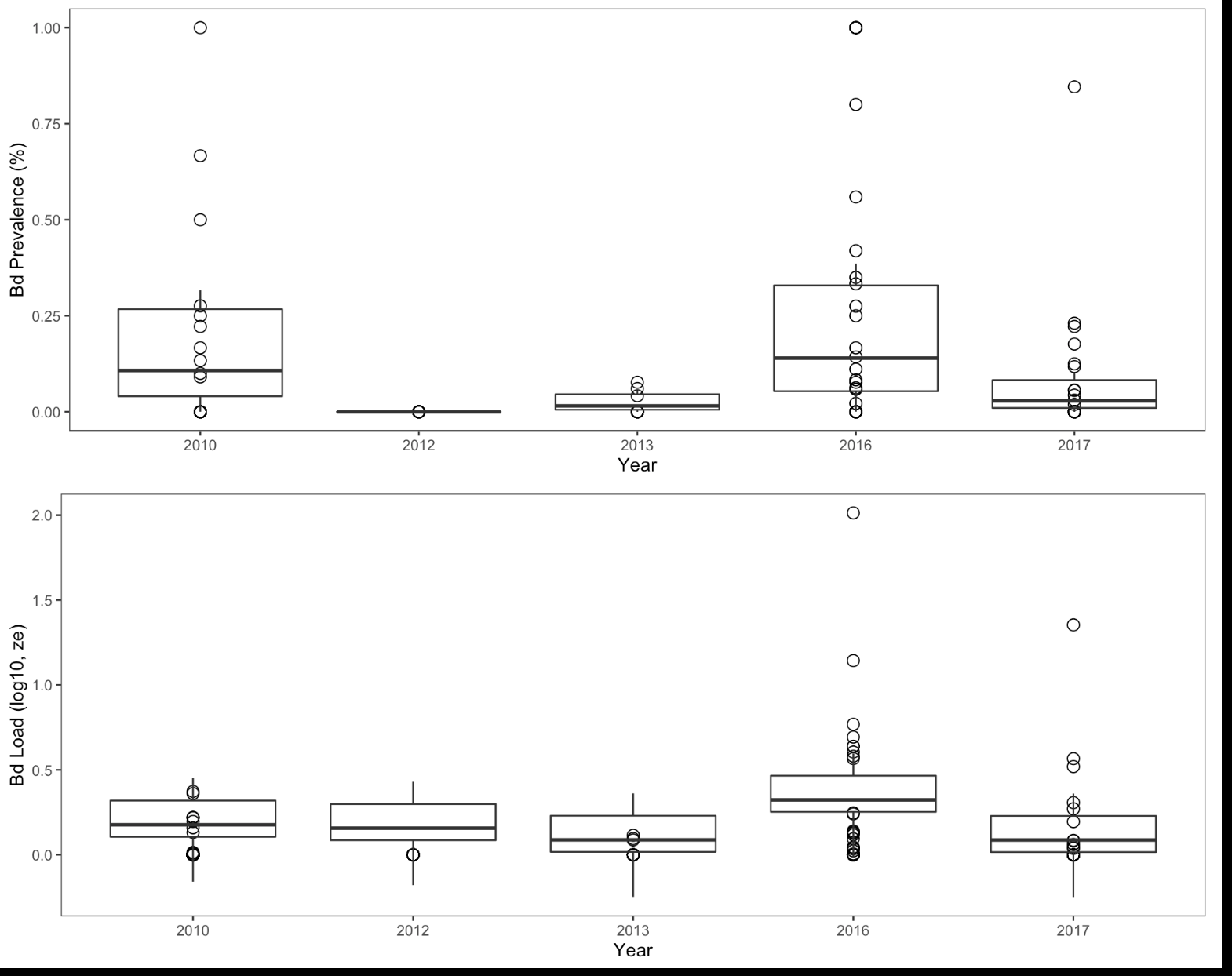


Supplementary Figure 1: Predicted values (boxplot) for *Bd* prevalence (top panel) and *Bd* load (bottom panel) by year. Raw values (points) of *Bd* prevalence and *Bd* loads overlaid.

Supplementary figure 2: Results of the indicator species analysis (LEfSe) on ASV level for *Hylarana latouchii* (A) and *Nidirana adenopleura* (B). Copyright photos: Dirk S. Schmeller, Chun-Fu Lin.

Supplementary Table 1: Samples obtained (N = 2517) in the field. N = number of sampled individuals, Prevalence = proportion of infected individuals to the full sample, N *Bd*+ = number of *Bd*-positive samples, Mean ZE = Mean zoospore equivalents per species and their standard error (SE). A constantly updated list of species can be found at amphibia web (https://tinyurl.com/y2jnro7a). GPS coordinates of the different locations have not been given to protect rare and endemic species. * Taxonomic uncertainty, maximum upper elevation reported in IUCN Red list deviates from own observations.

| **Order** | **Family** | **Species** | **N field** | **Prevalence** | **N Bd+** | **Mean ZE** | **SE** | **Endemicity** | **IUCN 2017** | **lower elevation** | **upper elevation** |
| --- | --- | --- | --- | --- | --- | --- | --- | --- | --- | --- | --- |
| Anura | Bufonidae | *Bufo bankorensis* | 186 | 2.688 | 5 | 0.256 | 0.143 | endemic | LC | 10 | 2590 |
|  |  | *Duttaphrynus melanostictus* | 43 | 2.326 | 1 | 0.113 | 0.113 | not endemic | LC | 0 | 570 |
|  | Dicroglossidae | *Fejervarya limnocharis* | 50 | 2.000 | 1 | 0.079 | 0.079 | not endemic | LC | 0 | 1100 |
|  |  | *Hoplobatrachus rugulosus* | 104 |  |  |  |  | not endemic | LC | 5 | 570 |
|  |  | *Limnonectes fujianensis* | 126 | 3.175 | 4 | 131.679 | 130.948 | not endemic | LC | 50 | 1100 |
|  | Hylidae | *Hyla chinensis** | 79 |  |  |  |  | not endemic | LC | 10 | 570 (1000)* |
|  | Microhylidae | *Microhyla butleri* | 2 | 50.000 | 1 | 2.246 | 2.246 | not endemic | LC | 70 | 550 |
|  |  | *Microhyla fissipes* | 5 |  |  |  |  | not endemic | LC | 10 | 1100 |
|  |  | *Microhyla heymonsi* | 54 |  |  |  |  | not endemic | LC | 20 | 1100 |
|  | Ranidae | *Nidirana adenopleura* | 496 | 4.435 | 22 | 13.318 | 8.667 | not endemic | LC | 50 | 1200 |
|  |  | *Nidirana okinavana* | 43 | 4.651 | 2 | 0.587 | 0.558 | not endemic | EN | 560 | 842 |
|  |  | *Sylvirana guentheri* | 2 |  |  |  |  | not endemic | LC | 10 | 600 |
|  |  | *Hylarana latouchii* | 655 | 12.061 | 79 | 158.784 | 137.834 | not endemic | LC | 10 | 1200 |
|  |  | *Lithobates catesbeianus* | 60 |  |  |  |  | alien | LC | 15 | 780 |
|  |  | *Odorrana swinhoana* | 40 |  |  |  |  | endemic | LC | 20 | 2260 |
|  |  | *Rana sauteri* | 34 | 5.882 | 2 | 0.907 | 0.882 | endemic | EN | 50 | 3100 |
|  |  | *Rana longicrus* | 21 |  |  |  |  | not endemic | VU | 10 | 890 |
|  | Rhacophoridae | *Buergeria choui* | 73 | 47.945 | 35 | 101.738 | 24.705 | not endemic | LC | 20 | 1170 |
|  |  | *Buergeria robusta* | 34 | 8.824 | 3 | 22.941 | 16.745 | endemic | LC | 20 | 1100 |
|  |  | *Kurixalus eiffingeri* | 20 | 10.000 | 2 | 4.401 | 4.400 | not endemic | LC | 140 | 2500 |
|  |  | *Kurixalus idiootocus* | 81 | 2.469 | 2 | 0.020 | 0.018 | endemic | LC | 10 | 1205 |
|  |  | *Polypedates braueri* | 61 |  |  |  |  | not endemic | LC | 20 | 1100 |
|  |  | *Zhangixalus moltrechti* | 117 | 14.530 | 17 | 4.678 | 1.591 | endemic | LC | 100 | 2500 |
| **Caudata** | Hynobiidae | *Hynobius arisanensis* | 31 | 3.226 | 1 | 0.044 | 0.044 | endemic | VU | 2260 | 3580 |
|  |  | *Hynobius formosanus* | 20 | 5.000 | 1 | 0.064 | 0.064 | endemic | EN | 2300 | 2900 |
|  |  | *Hynobius sonani* | 80 | 5.000 | 4 | 6.163 | 5.433 | endemic | EN | 2500 | 3100 |
|  |  |  |  |  |  |  |  |  |  |  |  |

Supplementary table 2: Sample description of the museum samples, ZE = Zoospore Equivalents, NTNU = National Taiwan Normal University, NSM = Natural Science Museum Taichung.

| **Collection  ID #** | **Order** | **Family** | **Species Name** | **Infection status** | **ZE** | **Collection Date** | **Year** | **Period** | **GPS - North** | **GPS - East** | **Elevation (m)** | **Collection Site Name** |
| --- | --- | --- | --- | --- | --- | --- | --- | --- | --- | --- | --- | --- |
| 54601 | Caudata | Hynobiidae | *Hynobius arisanensis* | Bd- | 0 | 25/08/1977 | 1981 | 1980-1989 |  |  |  | NTNU |
| 54604 | Caudata | Hynobiidae | *Hynobius arisanensis* | Bd- | 0 | 25/08/1977 | 1981 | 1980-1989 |  |  |  | NTNU |
| 54502 | Caudata | Hynobiidae | *Hynobius arisanensis* | Bd- | 0 | 29/08/1977 | 1981 | 1980-1989 |  |  |  | NTNU |
| 165301 | Caudata | Hynobiidae | *Hynobius arisanensis* | Bd- | 0 | 23/03/1978 | 1982 | 1980-1989 |  |  |  | NTNU |
| 163103 | Caudata | Hynobiidae | *Hynobius arisanensis* | Bd- | 0 | 07/05/1978 | 1982 | 1980-1989 |  |  |  | NTNU |
| 163104 | Caudata | Hynobiidae | *Hynobius arisanensis* | Bd- | 0 | 07/05/1978 | 1982 | 1980-1989 |  |  |  | NTNU |
| 163503 | Caudata | Hynobiidae | *Hynobius sp* | Bd- | 0 | 28/07/1978 | 1982 | 1980-1989 |  |  |  | NTNU |
| 163509 | Caudata | Hynobiidae | *Hynobius sp* | Bd- | 0 | 29/07/1978 | 1982 | 1980-1989 |  |  |  | NTNU |
| 163701 | Caudata | Hynobiidae | *Hynobius arisanensis* | Bd- | 0 | 23/08/1978 | 1982 | 1980-1989 |  |  |  | NTNU |
| 163902 | Caudata | Hynobiidae | *Hynobius arisanensis* | Bd- | 0 | 25/08/1978 | 1982 | 1980-1989 |  |  |  | NTNU |
| 163903 | Caudata | Hynobiidae | *Hynobius arisanensis* | Bd- | 0 | 25/08/1978 | 1982 | 1980-1989 |  |  |  | NTNU |
| 164103 | Caudata | Hynobiidae | *Hynobius arisanensis* | Bd- | 0 | 28/08/1978 | 1982 | 1980-1989 |  |  |  | NTNU |
| 164102 | Caudata | Hynobiidae | *Hynobius arisanensis* | Bd- | 0 | 28/08/1978 | 1982 | 1980-1989 |  |  |  | NTNU |
| 164201 | Caudata | Hynobiidae | *Hynobius arisanensis* | Bd- | 0 | 08/09/1978 | 1982 | 1980-1989 |  |  |  | NTNU |
| 164202 | Caudata | Hynobiidae | *Hynobius arisanensis* | Bd- | 0 | 08/09/1978 | 1982 | 1980-1989 |  |  |  | NTNU |
| 165002 | Caudata | Hynobiidae | *Hynobius arisanensis* | Bd- | 0 | 20/01/1980 | 1984 | 1980-1989 |  |  |  | NTNU |
| 165001 | Caudata | Hynobiidae | *Hynobius arisanensis* | Bd- | 0 | 20/01/1980 | 1984 | 1980-1989 |  |  |  | NTNU |
| 165204 | Caudata | Hynobiidae | *Hynobius arisanensis* | Bd- | 0 | 06/03/1980 | 1984 | 1980-1989 |  |  |  | NTNU |
| 165205 | Caudata | Hynobiidae | *Hynobius arisanensis* | Bd- | 0 | 06/03/1980 | 1984 | 1980-1989 |  |  |  | NTNU |
| 165302 | Caudata | Hynobiidae | *Hynobius arisanensis* | Bd- | 0 | 23/03/1980 | 1984 | 1980-1989 |  |  |  | NTNU |
| 49 | Anura | Rhacophoridae | *Zhangixalus moltrechti* | Bd- | 0 | 17/04/1982 | 1986 | 1980-1989 | 233104N | 1204824E | 2274 | NSM |
| 49 | Anura | Rhacophoridae | *Zhangixalus moltrechti* | Bd- | 0 | 17/04/1982 | 1986 | 1980-1989 | 233104N | 1204824E | 2274 | NSM |
| 78 | Anura | Ranidae | *Hylarana latouchii* | Bd- | 0 | 24/04/1982 | 1986 | 1980-1989 | 233021N | 1204114E | 1405 | NSM |
| 78 | Anura | Ranidae | *Hylarana latouchii* | Bd- | 0 | 24/04/1982 | 1986 | 1980-1989 | 233021N | 1204114E | 1405 | NSM |
| 78 | Anura | Ranidae | *Hylarana latouchii* | Bd- | 0 | 24/04/1982 | 1986 | 1980-1989 | 233021N | 1204114E | 1405 | NSM |
| 78 | Anura | Ranidae | *Hylarana latouchii* | Bd- | 0 | 24/04/1982 | 1986 | 1980-1989 | 233021N | 1204114E | 1405 | NSM |
| 77 | Anura | Rhacophoridae | *Zhangixalus moltrechti* | Bd- | 0 | 24/04/1982 | 1986 | 1980-1989 | 233020N | 1204114E | 1405 | NSM |
| 77 | Anura | Rhacophoridae | *Zhangixalus moltrechti* | Bd- | 0 | 24/04/1982 | 1986 | 1980-1989 | 233020N | 1204114E | 1405 | NSM |
| 77 | Anura | Rhacophoridae | *Zhangixalus moltrechti* | Bd- | 0 | 24/04/1982 | 1986 | 1980-1989 | 233020N | 1204114E | 1405 | NSM |
| 99 | Anura | Ranidae | *Hylarana latouchii* | Bd- | 0 | 02/06/1982 | 1986 | 1980-1989 | 240130N | 1210742E | 1148 | NSM |
| 99 | Anura | Ranidae | *Hylarana latouchii* | Bd- | 0 | 02/06/1982 | 1986 | 1980-1989 | 240130N | 1210742E | 1148 | NSM |
| 99 | Anura | Ranidae | *Hylarana latouchii* | Bd- | 0 | 02/06/1982 | 1986 | 1980-1989 | 240130N | 1210742E | 1148 | NSM |
| 99 | Anura | Ranidae | *Hylarana latouchii* | Bd- | 0 | 02/06/1982 | 1986 | 1980-1989 | 240130N | 1210742E | 1148 | NSM |
| 380 | Anura | Rhacophoridae | *Zhangixalus moltrechti* | Bd- | 0 | 24/05/1985 | 1989 | 1980-1989 | 233104N | 1204824E | 2274 | NSM |
| 376 | Anura | Rhacophoridae | *Zhangixalus moltrechti* | Bd- | 0 | 08/02/1986 | 1990 | 1990-1999 | 233020N | 1204114E | 1405 | NSM |
| 1241 | Anura | Ranidae | *Rana sauteri* | Bd- | 0 | 25/10/1986 | 1990 | 1990-1999 | 2337--N | 12045--E | 1600 | NSM |
| 1241 | Anura | Ranidae | *Rana sauteri* | Bd- | 0 | 25/10/1986 | 1990 | 1990-1999 | 2337--N | 12045--E | 1600 | NSM |
| 1241 | Anura | Ranidae | *Rana sauteri* | Bd- | 0 | 25/10/1986 | 1990 | 1990-1999 | 2337--N | 12045--E | 1600 | NSM |
| 1241 | Anura | Ranidae | *Rana sauteri* | Bd- | 0 | 25/10/1986 | 1990 | 1990-1999 | 2337--N | 12045--E | 1600 | NSM |
| 1241 | Anura | Ranidae | *Rana sauteri* | Bd- | 0 | 25/10/1986 | 1990 | 1990-1999 | 2337--N | 12045--E | 1600 | NSM |
| 1241 | Anura | Ranidae | *Rana sauteri* | Bd- | 0 | 25/10/1986 | 1990 | 1990-1999 | 2337--N | 12045--E | 1600 | NSM |
| 1241 | Anura | Ranidae | *Rana sauteri* | Bd- | 0 | 25/10/1986 | 1990 | 1990-1999 | 2337--N | 12045--E | 1600 | NSM |
| 1241 | Anura | Ranidae | *Rana sauteri* | Bd- | 0 | 25/10/1986 | 1990 | 1990-1999 | 2337--N | 12045--E | 1600 | NSM |
| 1241 | Anura | Ranidae | *Rana sauteri* | Bd- | 0 | 25/10/1986 | 1990 | 1990-1999 | 2337--N | 12045--E | 1600 | NSM |
| 1241 | Anura | Ranidae | *Rana sauteri* | Bd- | 0 | 25/10/1986 | 1990 | 1990-1999 | 2337--N | 12045--E | 1600 | NSM |
| 1241 | Anura | Ranidae | *Rana sauteri* | Bd- | 0 | 25/10/1986 | 1990 | 1990-1999 | 2337--N | 12045--E | 1600 | NSM |
| 1241 | Anura | Ranidae | *Rana sauteri* | Bd- | 0 | 25/10/1986 | 1990 | 1990-1999 | 2337--N | 12045--E | 1600 | NSM |
| 1241 | Anura | Ranidae | *Rana sauteri* | Bd- | 0 | 25/10/1986 | 1990 | 1990-1999 | 2337--N | 12045--E | 1600 | NSM |
| 1241 | Anura | Ranidae | *Rana sauteri* | Bd- | 0 | 25/10/1986 | 1990 | 1990-1999 | 2337--N | 12045--E | 1600 | NSM |
| 1241 | Anura | Ranidae | *Rana sauteri* | Bd- | 0 | 25/10/1986 | 1990 | 1990-1999 | 2337--N | 12045--E | 1600 | NSM |
| 4392 | Anura | Ranidae | *Rana sauteri* | Bd- | 0 | 25/10/1986 | 1990 | 1990-1999 | 2337--N | 12045--E | 1600 | NSM |
| 4392 | Anura | Ranidae | *Rana sauteri* | Bd- | 0 | 25/10/1986 | 1990 | 1990-1999 | 2337--N | 12045--E | 1600 | NSM |
| 4392 | Anura | Ranidae | *Rana sauteri* | Bd- | 0 | 25/10/1986 | 1990 | 1990-1999 | 2337--N | 12045--E | 1600 | NSM |
| 4392 | Anura | Ranidae | *Rana sauteri* | Bd- | 0 | 25/10/1986 | 1990 | 1990-1999 | 2337--N | 12045--E | 1600 | NSM |
| 4392 | Anura | Ranidae | *Rana sauteri* | Bd- | 0 | 25/10/1986 | 1990 | 1990-1999 | 2337--N | 12045--E | 1600 | NSM |
| 4392 | Anura | Ranidae | *Rana sauteri* | Bd+ | 0.904 | 25/10/1986 | 1990 | 1990-1999 | 2337--N | 12045--E | 1600 | NSM |
| 48 | Anura | Ranidae | *Rana sauteri* | Bd- | 0 | 25/10/1986 | 1990 | 1990-1999 | 233104N | 1204824E | 2274 | NSM |
| 48 | Anura | Ranidae | *Rana sauteri* | Bd- | 0 | 25/10/1986 | 1990 | 1990-1999 | 233104N | 1204824E | 2274 | NSM |
| 48 | Anura | Ranidae | *Rana sauteri* | Bd- | 0 | 25/10/1986 | 1990 | 1990-1999 | 233104N | 1204824E | 2274 | NSM |
| 48 | Anura | Ranidae | *Rana sauteri* | Bd- | 0 | 25/10/1986 | 1990 | 1990-1999 | 233104N | 1204824E | 2274 | NSM |
| 48 | Anura | Ranidae | *Rana sauteri* | Bd- | 0 | 25/10/1986 | 1990 | 1990-1999 | 233104N | 1204824E | 2274 | NSM |
| 48 | Anura | Ranidae | *Rana sauteri* | Bd- | 0 | 25/10/1986 | 1990 | 1990-1999 | 233104N | 1204824E | 2274 | NSM |
| 48 | Anura | Ranidae | *Rana sauteri* | Bd- | 0 | 25/10/1986 | 1990 | 1990-1999 | 233104N | 1204824E | 2274 | NSM |
| 48 | Anura | Ranidae | *Rana sauteri* | Bd- | 0 | 25/10/1986 | 1990 | 1990-1999 | 233104N | 1204824E | 2274 | NSM |
| 48 | Anura | Ranidae | *Rana sauteri* | Bd- | 0 | 25/10/1986 | 1990 | 1990-1999 | 233104N | 1204824E | 2274 | NSM |
| 48 | Anura | Ranidae | *Rana sauteri* | Bd- | 0 | 25/10/1986 | 1990 | 1990-1999 | 233104N | 1204824E | 2274 | NSM |
| 48 | Anura | Ranidae | *Rana sauteri* | Bd- | 0 | 25/10/1986 | 1990 | 1990-1999 | 233104N | 1204824E | 2274 | NSM |
| 48 | Anura | Ranidae | *Rana sauteri* | Bd- | 0 | 25/10/1986 | 1990 | 1990-1999 | 233104N | 1204824E | 2274 | NSM |
| 1700 | Anura | Ranidae | *Hylarana latouchii* | Bd- | 0 | 11/09/1987 | 1991 | 1990-1999 | 2401--N | 12108--E | 1120 | NSM |
| 1700 | Anura | Ranidae | *Hylarana latouchii* | Bd- | 0 | 11/09/1987 | 1991 | 1990-1999 | 2401--N | 12108--E | 1120 | NSM |
| 163401 | Caudata | Hynobiidae | *Hynobius arisanensis* | Bd- | 0 | 10/07/1988 | 1992 | 1990-1999 |  |  |  | NTNU |
| 201619 | Caudata | Hynobiidae | *Hynobius arisanensis* | Bd- | 0 | 07/07/1989 | 1993 | 1990-1999 |  |  |  | NTNU |
| 201627 | Caudata | Hynobiidae | *Hynobius arisanensis* | Bd- | 0 | 07/07/1989 | 1993 | 1990-1999 |  |  |  | NTNU |
| 201620 | Caudata | Hynobiidae | *Hynobius arisanensis* | Bd- | 0 | 07/07/1989 | 1993 | 1990-1999 |  |  |  | NTNU |
| 201616 | Caudata | Hynobiidae | *Hynobius arisanensis* | Bd- | 0 | 07/07/1989 | 1993 | 1990-1999 |  |  |  | NTNU |
| 201621 | Caudata | Hynobiidae | *Hynobius arisanensis* | Bd- | 0 | 07/07/1989 | 1993 | 1990-1999 |  |  |  | NTNU |
| 201742 | Caudata | Hynobiidae | *Hynobius sp.* | Bd- | 0 | 31/08/1989 | 1993 | 1990-1999 |  |  |  | NTNU |
| 201680 | Caudata | Hynobiidae | *Hynobius formosanus* | Bd- | 0 | 01/01/1990 | 1994 | 1990-1999 |  |  |  | NTNU |
| 201679 | Caudata | Hynobiidae | *Hynobius formosanus* | Bd- | 0 | 01/01/1990 | 1994 | 1990-1999 |  |  |  | NTNU |
| 201628 | Caudata | Hynobiidae | *Hynobius arisanensis* | Bd- | 0 | 11/03/1990 | 1994 | 1990-1999 |  |  |  | NTNU |
| 201681 | Caudata | Hynobiidae | *Hynobius formosanus* | Bd- | 0 | 29/06/1990 | 1994 | 1990-1999 |  |  |  | NTNU |
| 201682 | Caudata | Hynobiidae | *Hynobius formosanus* | Bd+ | 1.3840 | 29/06/1990 | 1994 | 1990-1999 |  |  |  | NTNU |
| 201683 | Caudata | Hynobiidae | *Hynobius formosanus* | Bd- | 0 | 29/06/1990 | 1994 | 1990-1999 |  |  |  | NTNU |
| 201685 | Caudata | Hynobiidae | *Hynobius formosanus* | Bd- | 0 | 29/06/1990 | 1994 | 1990-1999 |  |  |  | NTNU |
| 201636 | Caudata | Hynobiidae | *Hynobius arisanensis* | Bd- | 0 | 09/07/1990 | 1994 | 1990-1999 |  |  |  | NTNU |
| 201637 | Caudata | Hynobiidae | *Hynobius arisanensis* | Bd- | 0 | 09/07/1990 | 1994 | 1990-1999 |  |  |  | NTNU |
| 201642 | Caudata | Hynobiidae | *Hynobius arisanensis* | Bd- | 0 | 09/07/1990 | 1994 | 1990-1999 |  |  |  | NTNU |
| 201638 | Caudata | Hynobiidae | *Hynobius arisanensis* | Bd- | 0 | 09/07/1990 | 1994 | 1990-1999 |  |  |  | NTNU |
| 201712 | Caudata | Hynobiidae | *Hynobius sonani* | Bd- | 0 | 20/07/1990 | 1994 | 1990-1999 |  |  |  | NTNU |
| 201711 | Caudata | Hynobiidae | *Hynobius sonani* | Bd- | 0 | 20/07/1990 | 1994 | 1990-1999 |  |  |  | NTNU |
| 201718 | Caudata | Hynobiidae | *Hynobius sonani* | Bd- | 0 | 26/07/1990 | 1994 | 1990-1999 |  |  |  | NTNU |
| 201716 | Caudata | Hynobiidae | *Hynobius sonani* | Bd- | 0 | 26/07/1990 | 1994 | 1990-1999 |  |  |  | NTNU |
| 201720 | Caudata | Hynobiidae | *Hynobius sonani* | Bd- | 0 | 26/07/1990 | 1994 | 1990-1999 |  |  |  | NTNU |
| 201719 | Caudata | Hynobiidae | *Hynobius sonani* | Bd- | 0 | 26/07/1990 | 1994 | 1990-1999 |  |  |  | NTNU |
| -- | Anura | Ranidae | *Rana sauteri* | Bd- | 0 | 28/09/1990 | 1994 | 1990-1999 | 234024N | 1204721E | 1100 | NSM |
| -- | Anura | Ranidae | *Rana sauteri* | Bd- | 0 | 28/09/1990 | 1994 | 1990-1999 | 234024N | 1204721E | 1100 | NSM |
| 2543 | Anura | Ranidae | *Rana sauteri* | Bd- | 0 | 28/09/1990 | 1994 | 1990-1999 | 234024N | 1204721E | 1100 | NSM |
| 2543 | Anura | Ranidae | *Rana sauteri* | Bd- | 0 | 28/09/1990 | 1994 | 1990-1999 | 234024N | 1204721E | 1100 | NSM |
| 2543 | Anura | Ranidae | *Rana sauteri* | Bd- | 0 | 28/09/1990 | 1994 | 1990-1999 | 234024N | 1204721E | 1100 | NSM |
| 2543 | Anura | Ranidae | *Rana sauteri* | Bd- | 0 | 28/09/1990 | 1994 | 1990-1999 | 234024N | 1204721E | 1100 | NSM |
| 2543 | Anura | Ranidae | *Rana sauteri* | Bd- | 0 | 28/09/1990 | 1994 | 1990-1999 | 234024N | 1204721E | 1100 | NSM |
| 2543-6 | Anura | Ranidae | *Rana sauteri* | Bd- | 0 | 28/09/1990 | 1994 | 1990-1999 | 234024N | 1204721E | 1100 | NSM |
| 2543-15 | Anura | Ranidae | *Rana sauteri* | Bd- | 0 | 28/09/1990 | 1994 | 1990-1999 | 234024N | 1204721E | 1100 | NSM |
| 2543-10 | Anura | Ranidae | *Rana sauteri* | Bd- | 0 | 28/09/1990 | 1994 | 1990-1999 | 234024N | 1204721E | 1100 | NSM |
| 2543-11 | Anura | Ranidae | *Rana sauteri* | Bd- | 0 | 28/09/1990 | 1994 | 1990-1999 | 234024N | 1204721E | 1100 | NSM |
| 2543-14 | Anura | Ranidae | *Rana sauteri* | Bd- | 0 | 28/09/1990 | 1994 | 1990-1999 | 234024N | 1204721E | 1100 | NSM |
| 2543-12 | Anura | Ranidae | *Rana sauteri* | Bd- | 0 | 28/09/1990 | 1994 | 1990-1999 | 234024N | 1204721E | 1100 | NSM |
| 2543-8 | Anura | Ranidae | *Rana sauteri* | Bd- | 0 | 28/09/1990 | 1994 | 1990-1999 | 234024N | 1204721E | 1100 | NSM |
| 2543-13 | Anura | Ranidae | *Rana sauteri* | Bd- | 0 | 28/09/1990 | 1994 | 1990-1999 | 234024N | 1204721E | 1100 | NSM |
| 2543-7 | Anura | Ranidae | *Rana sauteri* | Bd- | 0 | 28/09/1990 | 1994 | 1990-1999 | 234024N | 1204721E | 1100 | NSM |
| 2543-9 | Anura | Ranidae | *Rana sauteri* | Bd- | 0 | 28/09/1990 | 1994 | 1990-1999 | 234024N | 1204721E | 1100 | NSM |
| 2717 | Anura | Ranidae | *Hylarana latouchii* | Bd- | 0 | 16/05/1991 | 1995 | 1990-1999 | 234024N | 1204721E | 1200 | NSM |
| 2717 | Anura | Ranidae | *Hylarana latouchii* | Bd- | 0 | 16/05/1991 | 1995 | 1990-1999 | 234024N | 1204721E | 1200 | NSM |
| 2717 | Anura | Ranidae | *Hylarana latouchii* | Bd- | 0 | 16/05/1991 | 1995 | 1990-1999 | 234024N | 1204721E | 1200 | NSM |
| 2717 | Anura | Ranidae | *Hylarana latouchii* | Bd- | 0 | 16/05/1991 | 1995 | 1990-1999 | 234024N | 1204721E | 1200 | NSM |
| 2717 | Anura | Ranidae | *Hylarana latouchii* | Bd- | 0 | 16/05/1991 | 1995 | 1990-1999 | 234024N | 1204721E | 1200 | NSM |
| 2717 | Anura | Ranidae | *Hylarana latouchii* | Bd- | 0 | 16/05/1991 | 1995 | 1990-1999 | 234024N | 1204721E | 1200 | NSM |
| 2717 | Anura | Ranidae | *Hylarana latouchii* | Bd- | 0 | 16/05/1991 | 1995 | 1990-1999 | 234024N | 1204721E | 1200 | NSM |
| 2717 | Anura | Ranidae | *Hylarana latouchii* | Bd- | 0 | 16/05/1991 | 1995 | 1990-1999 | 234024N | 1204721E | 1200 | NSM |
| 2717 | Anura | Ranidae | *Hylarana latouchii* | Bd- | 0 | 16/05/1991 | 1995 | 1990-1999 | 234024N | 1204721E | 1200 | NSM |
| 2717 | Anura | Ranidae | *Hylarana latouchii* | Bd- | 0 | 16/05/1991 | 1995 | 1990-1999 | 234024N | 1204721E | 1200 | NSM |
| 2717 | Anura | Ranidae | *Hylarana latouchii* | Bd- | 0 | 16/05/1991 | 1995 | 1990-1999 | 234024N | 1204721E | 1200 | NSM |
| 2717 | Anura | Ranidae | *Hylarana latouchii* | Bd- | 0 | 16/05/1991 | 1995 | 1990-1999 | 234024N | 1204721E | 1200 | NSM |
| 201653 | Caudata | Hynobiidae | *Hynobius arisanensis* | Bd- | 0 | 14/11/1995 | 1999 | 1990-1999 |  |  |  | NTNU |
| 201658 | Caudata | Hynobiidae | *Hynobius arisanensis* | Bd- | 0 | 14/11/1995 | 1999 | 1990-1999 |  |  |  | NTNU |
| 201659 | Caudata | Hynobiidae | *Hynobius arisanensis* | Bd- | 0 | 14/11/1995 | 1999 | 1990-1999 |  |  |  | NTNU |
| 201657 | Caudata | Hynobiidae | *Hynobius arisanensis* | Bd- | 0 | 14/11/1995 | 1999 | 1990-1999 |  |  |  | NTNU |
| 201650 | Caudata | Hynobiidae | *Hynobius arisanensis* | Bd- | 0 | 14/11/1995 | 1999 | 1990-1999 |  |  |  | NTNU |
| 201652 | Caudata | Hynobiidae | *Hynobius arisanensis* | Bd- | 0 | 14/11/1995 | 1999 | 1990-1999 |  |  |  | NTNU |
| 201654 | Caudata | Hynobiidae | *Hynobius arisanensis* | Bd- | 0 | 14/11/1995 | 1999 | 1990-1999 |  |  |  | NTNU |
| 201655 | Caudata | Hynobiidae | *Hynobius arisanensis* | Bd- | 0 | 14/11/1995 | 1999 | 1990-1999 |  |  |  | NTNU |
| 201656 | Caudata | Hynobiidae | *Hynobius arisanensis* | Bd- | 0 | 14/11/1995 | 1999 | 1990-1999 |  |  |  | NTNU |
| 201651 | Caudata | Hynobiidae | *Hynobius arisanensis* | Bd- | 0 | 14/11/1995 | 1999 | 1990-1999 |  |  |  | NTNU |
| 201744 | Caudata | Hynobiidae | *Hynobius sp.* | Bd- | 0 | 13/07/1996 | 2000 | 2000-2009 |  |  |  | NTNU |
| 201661 | Caudata | Hynobiidae | *Hynobius arisanensis* | Bd+ | 0.7816 | 11/01/1997 | 2001 | 2000-2009 |  |  |  | NTNU |
| 201660 | Caudata | Hynobiidae | *Hynobius arisanensis* | Bd- | 0 | 11/01/1997 | 2001 | 2000-2009 |  |  |  | NTNU |
| 201666 | Caudata | Hynobiidae | *Hynobius arisanensis* | Bd- | 0 | 12/02/1997 | 2001 | 2000-2009 |  |  |  | NTNU |
| 201675 | Caudata | Hynobiidae | *Hynobius arisanensis* | Bd+ | 1.4080 | 12/02/1997 | 2001 | 2000-2009 |  |  |  | NTNU |
| 201671 | Caudata | Hynobiidae | *Hynobius arisanensis* | Bd- | 0 | 12/02/1997 | 2001 | 2000-2009 |  |  |  | NTNU |
| 201663 | Caudata | Hynobiidae | *Hynobius arisanensis* | Bd- | 0 | 12/02/1997 | 2001 | 2000-2009 |  |  |  | NTNU |
| 201673 | Caudata | Hynobiidae | *Hynobius arisanensis* | Bd- | 0 | 12/02/1997 | 2001 | 2000-2009 |  |  |  | NTNU |
| 201667 | Caudata | Hynobiidae | *Hynobius arisanensis* | Bd- | 0 | 12/02/1997 | 2001 | 2000-2009 |  |  |  | NTNU |
| 201664 | Caudata | Hynobiidae | *Hynobius arisanensis* | Bd- | 0 | 12/02/1997 | 2001 | 2000-2009 |  |  |  | NTNU |
| 201662 | Caudata | Hynobiidae | *Hynobius arisanensis* | Bd- | 0 | 12/02/1997 | 2001 | 2000-2009 |  |  |  | NTNU |
| 201665 | Caudata | Hynobiidae | *Hynobius arisanensis* | Bd- | 0 | 12/02/1997 | 2001 | 2000-2009 |  |  |  | NTNU |
| 201672 | Caudata | Hynobiidae | *Hynobius arisanensis* | Bd- | 0 | 12/02/1997 | 2001 | 2000-2009 |  |  |  | NTNU |
| 201669 | Caudata | Hynobiidae | *Hynobius arisanensis* | Bd- | 0 | 12/02/1997 | 2001 | 2000-2009 |  |  |  | NTNU |
| 201668 | Caudata | Hynobiidae | *Hynobius arisanensis* | Bd- | 0 | 12/02/1997 | 2001 | 2000-2009 |  |  |  | NTNU |
| 201693 | Caudata | Hynobiidae | *Hynobius formosanus* | Bd- | 0 | 20/05/1997 | 2001 | 2000-2009 |  |  |  | NTNU |
| 201692 | Caudata | Hynobiidae | *Hynobius formosanus* | Bd- | 0 | 20/05/1997 | 2001 | 2000-2009 |  |  |  | NTNU |
| 201695 | Caudata | Hynobiidae | *Hynobius formosanus* | Bd- | 0 | 07/07/1997 | 2001 | 2000-2009 |  |  |  | NTNU |
| 201696 | Caudata | Hynobiidae | *Hynobius formosanus* | Bd- | 0 | 07/07/1997 | 2001 | 2000-2009 |  |  |  | NTNU |
| 201727 | Caudata | Hynobiidae | *Hynobius sonani* | Bd- | 0 | 27/07/1998 | 2002 | 2000-2009 |  |  |  | NTNU |
| 201746 | Caudata | Hynobiidae | *Hynobius sp.* | Bd- | 0 | 17/01/1999 | 2002 | 2000-2009 |  |  |  | NTNU |
| 201748 | Caudata | Hynobiidae | *Hynobius sp.* | Bd- | 0 | 07/07/1999 | 2003 | 2000-2009 |  |  |  | NTNU |
| 201697 | Caudata | Hynobiidae | *Hynobius formosanus* | Bd+ | 2 | 08/07/1999 | 2003 | 2000-2009 |  |  |  | NTNU |
| 201698 | Caudata | Hynobiidae | *Hynobius formosanus* | Bd- | 0 | 27/07/1999 | 2003 | 2000-2009 |  |  |  | NTNU |
| 201750 | Caudata | Hynobiidae | *Hynobius sp.* | Bd- | 0 | 29/02/2000 | 2004 | 2000-2009 |  |  |  | NTNU |
| 201749 | Caudata | Hynobiidae | *Hynobius sp.* | Bd- | 0 | 29/02/2000 | 2004 | 2000-2009 |  |  |  | NTNU |
| 3892 | Anura | Ranidae | *Hylarana latouchii* | Bd- | 0 | 26/03/2000 | 2004 | 2000-2009 | 234024N | 1204721E | 1120 | NSM |
| 3892 | Anura | Ranidae | *Hylarana latouchii* | Bd- | 0 | 26/03/2000 | 2004 | 2000-2009 | 234024N | 1204721E | 1120 | NSM |
| 3897 | Anura | Ranidae | *Hylarana latouchii* | Bd- | 0 | 02/04/2000 | 2004 | 2000-2009 | 234024N | 1204721E | 1120 | NSM |
| 3897 | Anura | Ranidae | *Hylarana latouchii* | Bd- | 0 | 02/04/2000 | 2004 | 2000-2009 | 234024N | 1204721E | 1120 | NSM |
| YF03 | Caudata | Hynobiidae | *Hynobius arisanensis* | Bd- | 0 | 15/08/2002 | 2006 | 2000-2009 | 120°57'22" | 23°27'25" | 3626 | NTNU |
| YF01 | Caudata | Hynobiidae | *Hynobius arisanensis* | Bd- | 0 | 15/08/2002 | 2006 | 2000-2009 | 120°57'22" | 23°27'25" | 3626 | NTNU |
| YF04 | Caudata | Hynobiidae | *Hynobius arisanensis* | Bd- | 0 | 15/08/2002 | 2006 | 2000-2009 | 120°57'22" | 23°27'25" | 3626 | NTNU |
| YF05 | Caudata | Hynobiidae | *Hynobius arisanensis* | Bd- | 0 | 15/08/2002 | 2006 | 2000-2009 | 120°57'22" | 23°27'25" | 3626 | NTNU |
| YF02 | Caudata | Hynobiidae | *Hynobius arisanensis* | Bd- | 0 | 15/08/2002 | 2006 | 2000-2009 | 120°57'22" | 23°27'25" | 3626 | NTNU |
| H0181 | Caudata | Hynobiidae | *Hynobius formosanus* | Bd- | 0 | 23/06/2005 | 2009 | 2000-2009 | 121°24'00" | 24°22'38" | 2500 | NTNU |
| TTC02 | Caudata | Hynobiidae | *Hynobius arisanensis* | Bd- | 0 | 28/06/2005 | 2009 | 2000-2009 | 120°53'31" | 23°28'44" | 2740 | NTNU |
| TTC01 | Caudata | Hynobiidae | *Hynobius arisanensis* | Bd- | 0 | 28/06/2005 | 2009 | 2000-2009 | 120°53'31" | 23°28'44" | 2740 | NTNU |

Supplementary Table 3:

| Variable | Type | Range | Abbreviation | Description |
| --- | --- | --- | --- | --- |
| Infection presence/absence | Binary | 0, 1 (n = 2090) | Prev | Probability of being infected, binary response variable: 0 = no infection detected, 1 = infection detected |
| Infection intensity | Numeric | 0 - 90,000 (n = 2090) | ZE_swab_ | Quantity of zoospores per individual swab based on qPCR results |
| Species | Categorical |  | S | 17 species used in models of 30 species sampled |
| Elevation | Continious | 0 - 3100 m | E | Elevation in meters above see level |
| Minimum temperature | Continious | 2.417 - 20.375 | T_min_ | yearly minimum temperature at focal sites |
| Mean temperature | Continious | 10.908 - 21.592 | T_mean_ | yearly mean temperature at focal sites |
| Maximum temperature | Continious | 9.467 - 26.692 | T_max_ | yearly maximum temperature at focal sites |
| Palmers Drought Severity Index | Continious | -1.3 - 3.075 | PDSI | measurement of dryness based on recent precipitation and temperature ^1^, yearly mean |
| Sample year | Categorical | 1981 - 2017 | Y | Field sampling in 2010 (n = 283), 2012 (n = 195), 2013 (n = 338), 2016 (n = 641), 2017 (n = 637), museum sampling (1981 - 2009) |
| Genus | Categorical |  | G | 11 genera were used in models; *Bufo* (n = 186), *Zhangixalus* (n = 117), *Rana* (n = 55), *Hylarana* (n = 655), *Buergeria* (n = 107), *Kurixalus* (n = 101), *Fejervarya* (n = 50), *Limnonectes* (n = 126), *Hynobius* (n = 131), *Duttaphrynus* (n = 43), *Nidirana* (n = 539) |

1 Palmer, W. C. *Meteorological drought*. Vol. 30 1-65 (US Department of Commerce, Weather Bureau, 1965).
